# Low phosphatase activity of LiaS and strong LiaR-DNA affinity explain the unusual LiaS to LiaR in vivo stoichiometry

**DOI:** 10.1186/s12866-020-01796-6

**Published:** 2020-04-29

**Authors:** Shailee Jani, Karen Sterzenbach, Vijaya Adatrao, Ghazal Tajbakhsh, Thorsten Mascher, Dasantila Golemi-Kotra

**Affiliations:** 1 Department of Biology, York University, Toronto, ON M3J1P3 Canada; 2 Institute for Microbiology, Technische Universität Dresden, Dresden, Germany

**Keywords:** Two-component system, LiaRS, Histidine kinase, Response regulator, *Bacillus subtilis*, Cell envelope stress

## Abstract

**Background:**

LiaRS mediates *Bacillus subtilis* response to cell envelope perturbations. A third protein, LiaF, has an inhibitory role over LiaRS in the absence of stimulus. Together, LiaF and LiaRS form a three-component system characterized by an unusual stoichiometry, a 4:1 ratio between LiaS and LiaR, the significance of which in the signal transduction mechanism of LiaRS is not entirely understood.

**Results:**

We measured, for the first time, the kinetics of the phosphorylation-dependent processes of LiaRS, the DNA-binding affinity of LiaR, and characterized the effect of phosphorylation on LiaR oligomerization state. Our study reveals that LiaS is less proficient as a phosphatase. Consequently, unspecific phosphorylation of LiaR by acetyl phosphate may be significant in vivo. This drawback is exacerbated by the strong interaction between LiaR and its own promoter, as it can drive LiaRS into losing grip over its own control in the absence of stimuli. These intrinsic, seemingly ‘disadvantageous”, attributes of LiaRS are likely overcome by the higher concentration of LiaS over LiaR in vivo, and a pro-phosphatase role of LiaF.

**Conclusions:**

Overall, our study shows that despite the conservative nature of two-component systems, they are, ultimately, tailored to meet specific cell needs by modulating the dynamics of interactions among their components and the kinetics of phosphorylation-mediated processes.

## Background

Two-component systems (TCS) represent a fundamental mechanism of bacterial signal transduction that allows microorganisms to perceive external signals and react appropriately with a cytoplasmic response. Typically, a two-component system (TCS) consists of a membrane bound (most of the time) histidine kinase (HK) and an intracellular soluble protein [1, 2]. The HK intercepts an environmental cue and, through an act of autophosphorylation, transduces the signal intracellularly [3, 4]. The response to the cue is mediated through a phosphotransfer process in which the second protein, referred to as the response regulator protein (RR), receives the phosphoryl group from the cognate HK at a conserved aspartate residue. However, studies have shown that the intracellular acetyl phosphate can also serve as a phosphodonor to RRs [5]. The phosphorylation of RR marks its activation and it is often associated with its dimerization [2]. Further, the function of RR, either a transcription factor (most of the time) or an enzyme, determines the outcome of the TCS signal-transduction pathway [6]. In the absence of an extracellular stimulus the signal transduction pathway is switched off through the phosphatase activity of HK. Despite the conservation of the HK and RR in TCS, each TCS performs differently.

The TCS LiaRS of *Bacillus subtilis* is involved in sensing cell envelope stress instigated by perturbation of the cytoplasmic membrane, particularly antibiotics that interfere with the Lipid II cycle of cell-wall peptidoglycan biosynthesis such as bacitracin, ramoplanin, vancomycin, and cationic antimicrobial peptides [7–12]. In addition, LiaRS is also induced by molecules that non-specifically disrupt the cytoplasmic membrane, such as detergents, ethanol, phenol, organic solvents and secretion stress [8, 13]. Gene deletion and mutagenesis studies showed that LiaS is involved in sensing cell envelope perturbation and that this HK is a bifunctional enzyme that possesses phosphatase activity in vivo. Furthermore, these studies showed that LiaR is susceptible to phosphorylation by acetyl phosphate in vivo: in the absence of *liaS* and at high *liaR* expression levels, this can lead to the activation of LiaR-dependent gene expression [14].

LiaRS is part of a three-component system, located in the *lia* locus of *B. subtilis*, in which a third protein, LiaF, acts as a strong inhibitor of LiaR-dependent gene expression in the absence of stimuli [15]. The trio, LiaRS-LiaF, is found in the same genomic context in many Gram-positive bacteria with a low G + C content (*Firmicutes*) [15]. The homologs of LiaRS TCS in *B. licheniformis*, *Streptococcus pneumonia*, *S. aureus*, *E. faecalis*, *E. faecium*, *L. monocytogens* and *S. mutans*, are also involved in the cell envelope stress response to bacitracin, vancomycin or cationic peptides [16–22]. The *lia* locus in *B. subtilis* is expressed from a strictly LiaR-dependent σ^A^-type promoter upstream of the *liaI* gene (the first of six genes in the *lia* locus) [8, 15], which represents the only relevant LiaR target in vivo [23]. This LiaR-dependent promoter is referred to as the *liaI* promoter (P_*liaI*_).

**Fig. 1:**
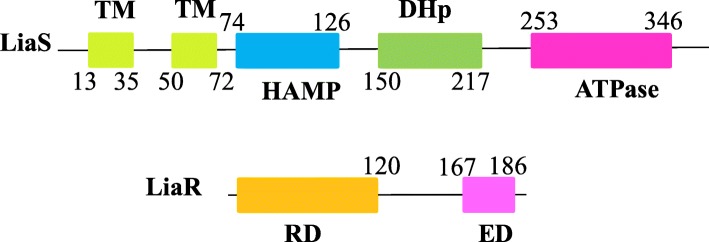
A diagram of domain organization in LiaS (ID:O32198) and LiaR (ID: O32197) as predicted by the Inter Proserver (EMBL-EBI). The “TM” symbols denote the transmembrane regions, “DHp” stands for dimerization and phosphotransfer domain, “HAMP” stands for histidine kinases, adenylyl cyclases, methyl-accepting chemotaxis proteins, and phosphatases, “RD” stands for receiver domain, and “ED” stands for effector domain

LiaS and LiaR are both modular proteins composed of a number of domains (Fig. 1). Analysis of the LiaS amino acid sequence by the UniProt server (ID O32198) revealed that this protein is a typical histidine kinase with two membrane-spanning regions, a HAMP domain, and an intracellular conserved histidine kinase region comprised of the dimerization and phosphotransfer domain (DHp; hosting the conserved histidine residue, His157) and the histidine kinase-like ATPase domain.

The two membrane-spanning regions in LiaS are connected with a very short extracellular linker that is characteristic of intramembrane-sensing HK (IM-HK). IM-HK are widely found in Gram-positive bacteria with a low G + C content (phylum *Firmicutes*) and are linked to the cell envelope stress response [7]. The lack of an extracellular domain connecting the two transmembrane regions in HK has led to the proposal that these HK may sense signals that arise either within or on the cytoplasmic membrane [9], and/or require auxiliary proteins to intercept extracellular signals [24].

The second transmembrane region of LiaS connects to the intracellular conserved histidine kinase region through a 52 amino acid long HAMP domain. The HAMP domains are found in histidine kinases, adenylyl cyclases, methyl-accepting chemotaxis proteins, and phosphatases. Studies have shown that the HAMP domains play a critical role in signal transduction across the transmembrane domains of these proteins to modulate the activity of their respective cytoplasmic domains [25, 26]. HAMP domains do not have much secondary structure on their own, an indication of possessing a highly flexible structure. However, they have propensity to fold into helix-turn-helix motifs when fused to transmembrane domains [27].

Analysis of the LiaR amino acid sequence by the UniProt server (ID O32197) revealed that LiaR is a typical two-domain RR that belongs to the FixJ/NarL family [28]. It is comprised of a conserved N-terminal domain, referred to as the Receiver domain (RD), that spans residues 3 to 119, and a variable C-terminal DNA-binding domain that spans the residues 143 to 208 (Fig. 1), referred to as the Effector domain (ED). LiaR ED belongs to the HTX-Lux family of DNA-binding proteins.

The biochemical details of LiaRS-dependent signal transduction remain poorly understood, particularly with regard to the interaction and phosphorylation between the HK LiaS and the RR LiaR. Remarkably, a genetic study demonstrated that the stoichiometry of LiaRS is crucial for its functionality: unlike many other TCSs, the HK is more abundant than the RR, with a native 4:1 ratio between LiaS and LiaR [14]. The relevance of this stoichiometry for the molecular mechanism of the signaling transduction by LiaRS has not been elucidated.

In this study, we undertook a biochemical approach to understand the signal transduction mechanism of LiaRS at the molecular level. The kinetics of phosphorylation-dependent pathways, that of LiaS autophosphorylation and LiaS-dependent phosphorylation of LiaR, were characterized and compared with the kinetics of LiaRS homologs in *S. aureus*, *S. mutans*, and *L. monocytogenes*. In addition, the kinetics of LiaS-dependent dephosphorylation of LiaR were studied to assess the efficiency of LiaS at a) switching off LiaRS-dependent signal transduction in the absence of stimuli, and b) guarding LiaR from activation by LiaS-independent phosphorylation pathways such as phosphorylation by acetyl phosphate. Furthermore, we probed the DNA-binding affinity of LiaR and assessed the role of phosphorylation in the DNA-binding activity of LiaR. Our studies provide the biochemical details for the mechanism of the LiaRS-dependent signal transduction pathway. Moreover they show that the uniqueness in performance by a TCS is determined by the dynamics of the interactions between the HK and RR proteins and the kinetics of the phosphorylation-transfer reactions that these proteins engage in.

## Results

### Purification of target proteins

In this study, LiaS and LiaSH159A variant were cloned fused to the C-terminus of the GST protein. While, LiaR, LiaR stand-alone domains, and LiaRD54A variant were cloned as tag-less proteins with no additional amino acids on either the N- or the C-termini of the proteins.

At first, we attempted to purify a LiaS variant that included the HAMP domain (the amino acid sequence spanning from 76 to 360). This LiaS variant was primarily trapped in the form of inclusion bodies, with a small amount of soluble protein available for purification. The purified protein did not exhibit autokinase activity. This could be due to the HAMP domain affecting the active state of the kinase or proper folding of the protein. We therefore cloned a version of LiaS that lacked the HAMP domain (the region from the amino acid 76 to 125). The production of this LiaS variant was sufficient and the protein primarily existed in a soluble form (Additional file 1: Figure S1A).

The purification of LiaR proved challenging, and we worked with a protein that was at least 85% pure as assessed by the coomassie staining of the SDS-PAGE (Additional file 1: Figure S1B). The LiaR^C^ and LiaR^N^ were purified to homogeneity as assessed by coomassie stained SDS-PAGE (Additional file 2: Figure S2). Based on the amino acid sequence of our target proteins, the calculated molecular masses of our purified proteins are: GST-LiaS 52,431 kD, LiaR 23,125 kD, LiaR^N^ 14,029 kD, and LiaR^C^ 8066 kD.

### Autophosphorylation activity of LiaS

**Fig. 2:**
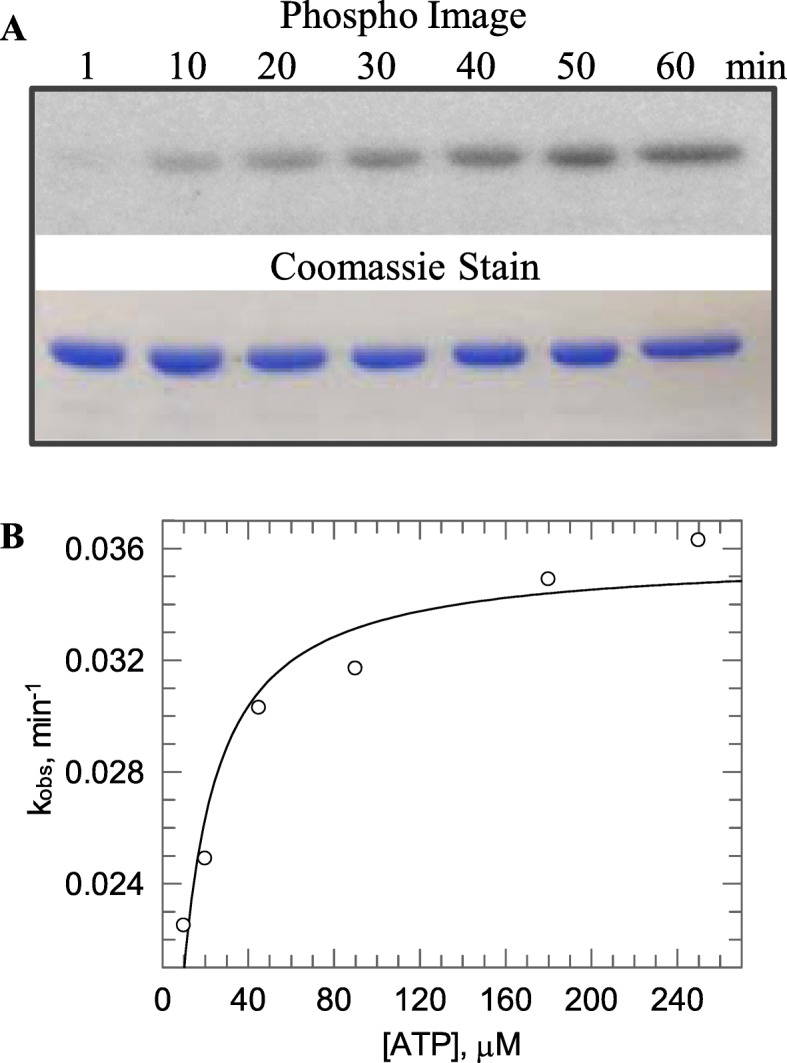
The autokinase activity of LiaS. **a** LiaS at 5 μM was incubated with 25 μM [γ-^32^P] ATP in PB at 25 °C. The reaction was quenched at different time intervals and samples were analyzed by a 12.5% SDS-PAGE. The SDS-PAGE was scanned with a phosphor screen to quantify the incorporation of [γ-^32^P] to LiaS (top pannel). The bottom panel is a coomassie stain of the SDS-PAGE to ensure equal loading of the samples. **b** The observed rate constants were plotted against ATP concentration to determine the *k*^app^ and *K*_S_

The kinetics of the LiaS autophosphorylation process was studied by incubating LiaS at 5 μM with different concentrations of [γ-^32^P]-ATP (10, 20, 45, 90, 180 and 250 μM), at 25 °C, and monitoring the incorporation of ^32^P isotope into LiaS (phosphorylation of LiaS) at different time intervals (Fig. 2a). The observed first-order rate constant of LiaS autophosphorylation (*k*_obs_) at a given ATP concentration was calculated by fitting the radiolabeled-protein bands on the phosphor-imaged gels to the equation *R*_t_ = *R*_max_ × (1-exp(−*k*_obs_*t*)) + *off* (Grafit software (version 5.0.10)), where *R*_t_ is the intensity of the radiolabeled-protein band at the time *t*, *R*_max_ is the maximum intensity reached on the protein band when all LiaS has undergone autophosphorylation, and *off* is the background signal observed on the phosphor-images of the gels; autophosphorylation of LiaS was exponential as reported for VraS, and not biphasic (Additional file 3: Figure S3A). The observed first-order rate constants were plotted against the ATP concentration to determine the apparent first-order rate constant of LiaS autophosphorylation (*k*^app^) and the dissociation constant (*K*_S_). The *k*^app^ value (rate constant) of LiaS was determined to be 0.036 ± 0.001 min^− 1^(at 25 °C), almost two-fold slower than *k*^app^ measured for VraS (0.07 min^− 1^). The *K*_S_ value was determined to be 7 ± 1 μM (Fig. 2b).

Phosphorylated LiaS was stable for up to 120 min. Substitution of the LiaS His159 residue for Ala abolished the autophosphorylation activity of LiaS (Additional file 3: Figure S3B), thus demonstrating that His159 is indeed the conserved phosphorylation site in LiaS.

The autophosphorylation rate constant of LiaS is comparable to the reported rate constants for other HKs, such as *E. coli* nitrate-responsive sensing HK NarQ (0.014 min^− 1^) [29], *S. aureus* cell membrane electrical potential sensing HK LytS (0.03 min^− 1^) [30], and *E. coli* citrate sensing HK DcuS (0.043 min^− 1^) [31]. Hence, LiaS is kinetically well suited to respond to cell envelope perturbations in *B. subtilis*. The binding affinity of LiaS for ATP is also comparable to other HK, such as LytS (7.9 μM) [30] and NarX (2.4 μM) [29], but it is higher than VraS (230 μM) [32], VanS (620 μM) [33] and *S. aureus* WalK (130 μM) [34].

### LiaR is the target of LiaS-kinase and -phosphatase activities, and of small-molecule phosphodonors

**Fig. 3:**
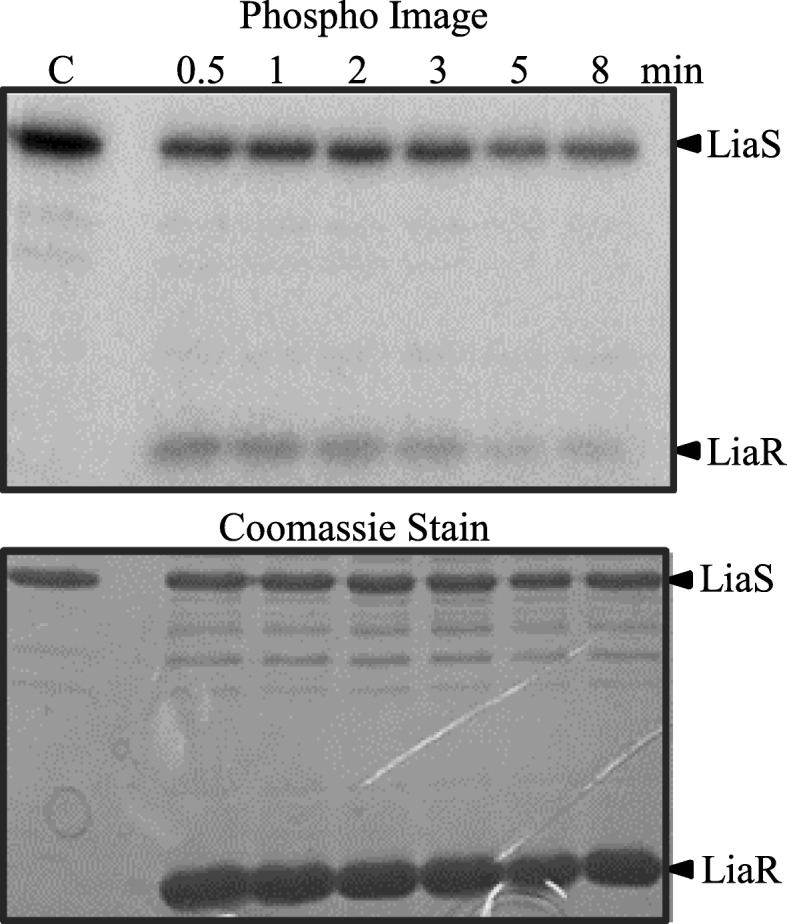
Phosphotransfer between LiaS and LiaR. Phosphorylated LiaS at 4 μM was incubated with 20 μM LiaR in PB at room temperature. The reaction was quenched at various time intervals and samples were analyzed by 15% SDS-PAGE. Gels were exposed to a phosphor screen (top panel) and imaged using a Typhoon Trio^+^ Imager (GE Healthcare), followed by coomassie staining (bottom panel)

A typical HK can rapidly transfer the phosphoryl group from its conserved histidine residue to a conserved aspartate residue of its cognate RR. Incubation of the phosphorylated LiaS with LiaR, led to a rapid transfer of the LiaS phosphoryl group to LiaR within 30 s (Fig. 3). These experiments show that LiaS is capable of phosphorylating its cognate RR. The observed first-order rate constant of the phosphotransfer process mediated by LiaS was estimated to be 1.39 min^− 1^, about four-fold slower in comparison to VraS (5.04 min^− 1^) [35], but ten-fold faster than LiaS from *Streptococcus mutans* (0.13 min^− 1^) [36].

The phosphotransfer experiments showed that LiaR gradually underwent dephosphorylation. The dephosphorylation process took about 8 min, and it was attributed to the LiaS phosphatase activity (Fig. 3). The rate constant of LiaS phosphatase activity against LiaR-P was estimated to be 0.08 min^− 1^, about ten-fold slower that the phosphatase activity of VraS against VraR-P (0.96 min^− 1^) [35]. No phosphatase activity has been reported for LiaS of *S. mutans* [36].

**Fig. 4:**
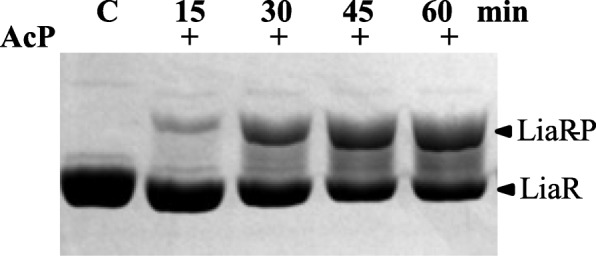
Phosphorylation of LiaR by acetyl phosphate. LiaR at 30 μM was incubated with 50 mM acetyl phosphate in PB at different times. The reaction was quenched by the addition of SDS-PAGE loading dye. Samples were analyzed by a 12.5% SDS-PAGE containing Phospho-TagT^M^. Gels were quantified by densitometry of bands using ImageJ

LiaS was not the only phosphodonor of the phosphoryl group to LiaR. We estimated the rate constant of LiaR phosphorylation by acetyl phosphate to be 0.032 ± 0.001 min^− 1^ (Fig. 4), slightly faster than the phosphorylation rate constant measured for VraR (0.022 min^− 1^) [35], and three-fold faster than LiaR of *S. mutans* (*Smt* LiaR; 0.011 min^− 1^) [36]. However, the autophosphorylation rate constant of LiaR is 20-fold slower than LytR (0.6 min^− 1^) [30], for which the phosphorylation by acetyl phosphate has been considered to be relevant in vivo [37]. Interestingly, RD of LiaR also underwent phosphorylation by acetyl phosphate, albeit at a much smaller yield (10%) and with a much smaller rate constant (Additional file 4: Figure S4). The large difference in the phosphorylation rate constants between full-length LiaR and stand-alone RD suggests a role of ED in holding RD in a conformational state that is more amenable to phosphorylation by acetyl phosphate.

When comparing the two independent phosphorylation processes of LiaR, we noted that LiaR phosphorylation by acetyl phosphate is 40-fold slower than its phosphorylation by LiaS. In the case of VraSR, the difference between these two phosphorylation-based processes is more pronounced, a 200-fold difference [35]. However, in the case of LiaRS of *S. mutans* (*Smt* LiaRS) this difference is ten-fold [36], and in the case of LytSR this difference is a mere two-fold [30]. In both these TCS, *Smt* LiaRS and LytSR, the phosphorylation of *Smt* LiaR and LytR by acetyl phosphate has been considered to be relevant in vivo [36, 37]. Thus, it is possible that the phosphorylation of *B. subtilis* LiaR (*Bsb* LiaR) by acetyl phosphate may be relevant in vivo, too.

Based on the sequence alignments to homologs of LiaR in *E. faecalis* (*Efc* LiaR), *E. faecium* (*Efm* LiaR), and *S. aureus* (VraR), the conserved phosphorylation site of LiaR was proposed to be Asp54. To confirm whether this residue is the phosphorylation site in LiaR, we substituted Asp54 for Ala. This substitution had no consequences to the secondary structural features of LiaR and its thermal stability as assessed by CD and thermal melting studies (Additional file 5: Figure S5). Instead, the substitution of Asp54 for Ala led to a protein that could not undergo phosphorylation (Additional file 6: Figure S6), thereby verifying its role as the acceptor site of LiaR for phosphotransfer*.*

### Oligomerization of LiaR

**Fig. 5:**
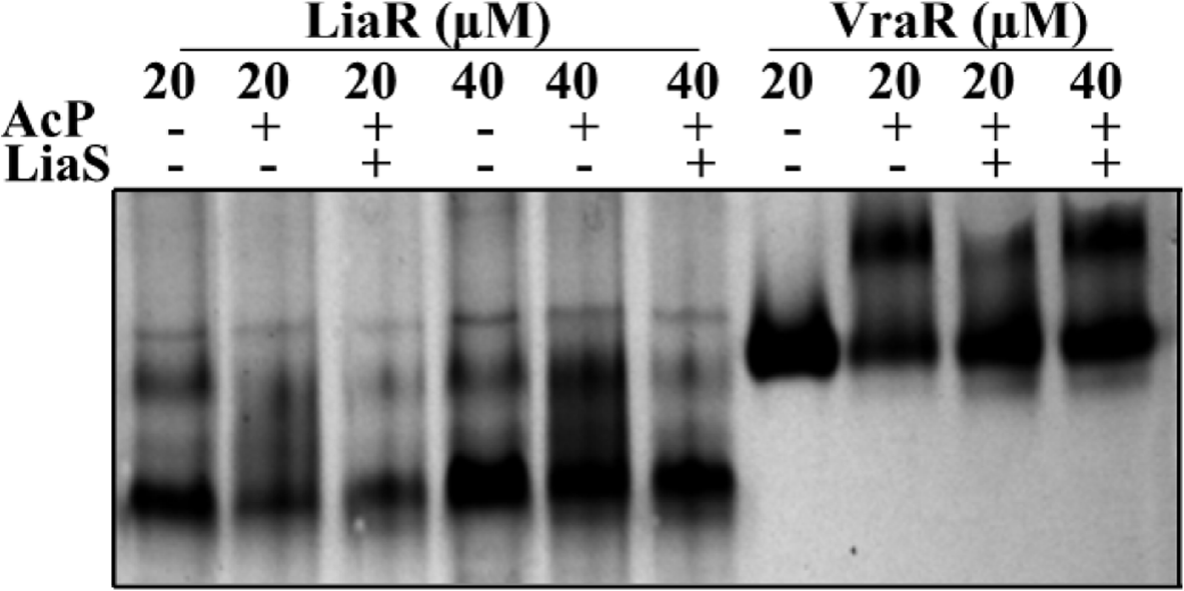
Oligomerization studies on LiaR. The oligomerization state of LiaR was analyzed by a 10% native-PAGE. Samples with or without acetyl phosphate (AcP) were incubated for 1 h in PB, at room temperature, and were resolved by gel electrophoresis. VraR was used as a control of the effect of phosphorylation in the oligomerization state of the protein. LiaS at 10 μM was added to investigate its phosphatase activity and its effect on the oligomerization of LiaR

The oligomerization state of LiaR was investigated by native-PAGE. These experiments showed that unphosphorylated LiaR existed as a mixture of monomer and dimer at concentrations of ≥20 μM (Fig. 5). These observations suggest that the dissociation constant for the dimer is > 20 μM. Phosphorylation of LiaR by acetyl phosphate led to a shift of this equilibrium toward the dimeric species. In the presence of LiaS, the equilibrium between monomeric and dimeric species was shifted toward monomeric species, thus confirming the phosphatase activity of LiaS, and the phosphorylation-dependence of the dimerization process (Fig. 5). The LiaR homologs in *E. faecalis*, *E. faecium* and *S. aureus* (VraR) behave differently; *Efm* LiaR and VraR are primarily monomeric in solution, and their respective dimeric species are initiated in a phosphorylation-dependent process, but *Efc* LiaR exists as a dimer in solution, with phosphorylation leading to the formation of a tetrameric species [35, 38, 39].

In this study, we cloned and purified RD and ED of LiaR to investigate the location of the dimerization interface. RD showed a clear partition of this domain between the monomeric and dimeric species at concentrations as low as 20 μM (Additional file 7: Figure S7). By contrast, ED existed as a monomer in solution as per the native-PAGE experiments, suggesting that LiaR undergoes dimerization at RD. The ED of *Efm* LiaR and *Efc* LiaR form strong dimers with a *K*_d_ value of 0.4 μM and 2 μM, respectively [38, 39], but no dimerization for ED of VraR was observed in solution [35].

### DNA-binding activity of LiaR

**Fig. 6:**
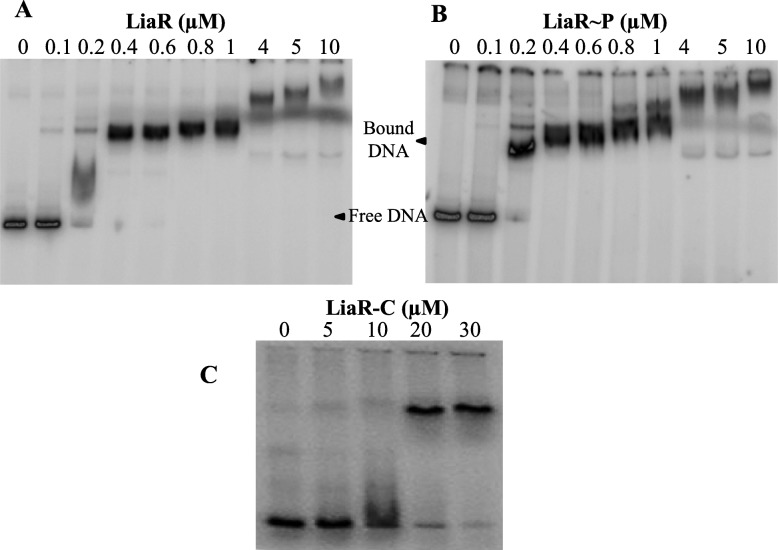
DNA-binding activity of LiaR investigated by EMSA. **a** LiaR was incubated with P_*liaI*_ at different concentrations. Samples were incubated for 30 min at room temperature. They were resolved in a 9% native-PAGE. The gels were exposed to a phosphor screen and imaged by a Typhoon Trio^+^ imager. **b** LiaR treated with acetyl phosphate, was incubated with P_*liaI*_ and analyzed as described above. **c** LiaR^C^ was incubated with P_*liaI*_ and analyzed as described above

The DNA-binding activity of LiaR was investigated by EMSA and DNase I footprinting. For the EMSA experiments, P_*liaI*_ spanning the nucleotides from − 162 to + 31 was amplified and labeled with ^32^P radioisotope on its 5′-ends. *P*_*liaI*_ was incubated with LiaR at concentrations that varied from 0.1 to 10 μM. The dissociation constant (*K*_d_) was estimated as the concentration of LiaR that bound 50% of the target DNA. The unphosphorylated LiaR protein bound with high binding affinity to *P*_*liaI*_, the *K*_d_ value was estimate to be 0.2 μM (Fig. 6a). By contrast, neither *Efm* LiaR nor *Efc* LiaR bound to their respective promoters in the unphosphorylated state [38, 39]. In the case of the LiaR homolog in *S. aureus*, VraR, it was shown that VraR interacted with the *vraSR* promoter in its unphosphorylated state (*K*_d_ = 5 μM), and phosphorylation of VraR increased the DNA-binding affinity 4 fold (*K*_d_ = 1.4 μM) [35, 40]. The comparison of the DNA-binding activities of *Bsb* LiaR to that of *Efm* LiaR or *Efc* LiaR provoked the question whether binding to DNA may have an effect on the oligomerization state of LiaR. Studies on VraR showed that the presence of DNA increased the VraR stability to trypsin digestion, likely by facilitating its dimerization (as a more stable structure) [35]. Hence, it is plausible that the presence of DNA may also contribute to the dimerization of LiaR.

Phosphorylation enhanced the LiaR DNA-binding affinity slightly, as assessed by EMSA (Fig. 6b). This could be due to the modest effect that phosphorylation has on the oligomerization state of LiaR, in our experimental setup (Fig. 5), or the limitations on the EMSA technique (see DNase I footprinting below). In the case of *Efm* LiaR and *Efc* LiaR, both proteins displayed no binding affinity to their target promoters in the unphosphorylated state. However, the DNA-binding affinities of their variants, respectively *Efm* LiaRW73C and *Efc* LiaRD191N (which represent fully activated LiaR proteins), for their target promoters were measured to be 13 μM and 0.88 μM (the phosphorylated or BeF^3−^ treated *Efm* LiaR and *Efc* LiaR were not assessed for their binding to their DNA target [38, 39]).

We investigated the significance of dimerization on the LiaR-binding activity by assessing the DNA-binding activity of its stand-alone ED. EMSA showed that ED bound to P_liaI_ 100-fold weaker than full-length LiaR (K_d_ (ED) ~ 20 μM) (Fig. 6c)*.* In the case of ED of Efm LiaR or Efc LiaR, no interaction was observed between ED and its respective DNA target. However, ED of VraR bound to its target DNA with a K_d_ of 7 μM (1.4-fold weaker than the full-length unphosphorylated VraR) [35, 38, 39]. The weak interaction between P_liaI_ and ED suggests that RD is essential to the DNA-binding activity of LiaR.

**Fig. 7:**
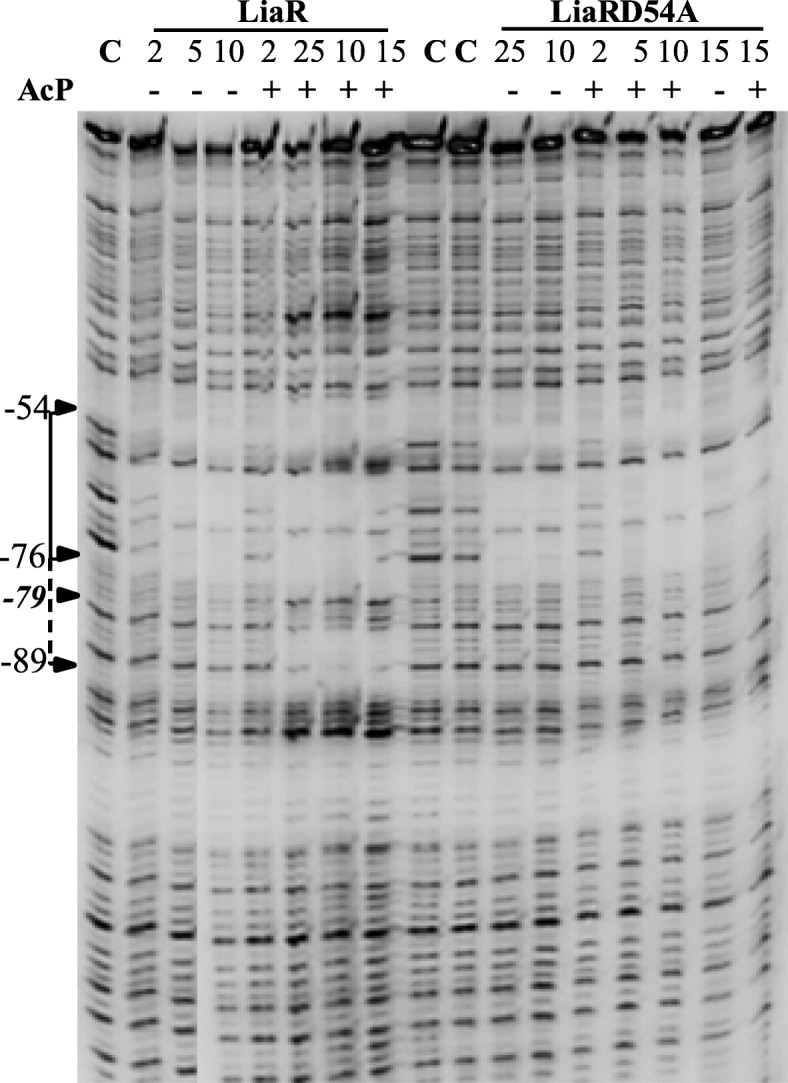
LiaR-DNase I footprint analysis of P_*liaI*_. The DNA fragment was labeled on the top strand at the 5′-end with [γ-^32^P]-ATP. The position of the protection site in the gel was determined from the running of the A, T, G and C standard reactions (Additional file 8: Figure S8). Protection of P_*liaI*_ by LiaR and LiaRD54A was investigated in the absence and presence of acetyl phosphate and at different protein concentrations. The primary binding site is indicated by a solid line, and the secondary binding site is indicated by a dashed line. The hypersensitive site at − 79 is indicated by an arrow

The DNA-binding activity of LiaR was also investigated by DNase I footprinting. These experiments showed that LiaR bound to the region from − 76 to − 52 of P_liaI_ (Fig. 7, Additional file 8: Figure S8). Moreover, the DNAse I footprinting experiments showed that phosphorylation expands the LiaR DNA-binding up to the − 89 position and it introduces a hypersensitive site at the − 79 position (a thymine). This hypersensitive site lies between the DNA-binding sites of LiaR (primary binding site) and LiaR ~ P (secondary binding site). To ensure that the LiaR secondary-binding site is linked to the phosphorylation of LiaR, we carried out the DNase I footprinting with the LiaRD54A variant. In these experiments the LiaRD54A variant was subjected to the same phosphorylation conditions as the wild-type protein. These studies showed that only wild-type LiaR accessed the secondary-binding site, when it was subjected to phosphorylation (Fig. 7).

**Fig. 8:**
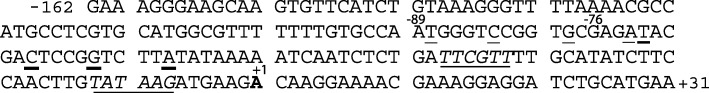
The DNA sequence of P_*liaI*_ used in the EMSA and DNase I footprinting studies. The conserved nucleotides in the LiaR-binding motifs are underlined. The primary LiaR binding site has the conserved nucleotides underlined with thicker lines. The − 35 and − 10 sequences are underlined and italicized. The transcription starting point (+ 1) is in bold

A closer inspection of the LiaR primary-binding site, the region from − 76 to − 52, revealed that this site hosts the LiaR-binding motif derived from a comparative genomics analysis, T(X)_4_C(X)_4_G(X)_4_A (Fig. 8). Moreover, this LiaR-binding site included the putative LiaR-binding sequence: ATAcGACtcccgGTCtTAT [15]. The putative LiaR-binding sequence was also identified in the target promoters of Efm LiaR, Efc LiaR, and LiaR from *Listeria monocytogenes* (Lmc LiaR) [21, 38, 39].

Investigation of the LiaR secondary-binding site, the region from − 89 to − 76, revealed the presence of an additional T(X)_4_C(X)_4_G(X)_4_A motif (Fig. 8). However, this region did not carry the putative LiaR-binding sequence, suggesting that it may interact weakly with LiaR, which is why it may require LiaR to be phosphorylated. A similar observation was made for VraR, which bound to a non-conserved secondary DNA-binding site only when it was phosphorylated [40]*.*

LiaR binding to a secondary DNA-binding site has also been reported for *Efm* LiaR and *Efc* LiaR [38, 39]. In these cases, the LiaR secondary-binding site was located either upstream or downstream of the primary-binding site, depending on the target promoter. The VraR secondary-binding site on the *vraSR* promoter was located downstream of the primary-binding site [38–40]. Hence, despite recognizing the same DNA-binding sequence, the LiaR homologs rely on different transcriptional regulation mechanisms depending on the organization of the DNA regulatory elements on their target promoters.

### Cross-talk between LiaRS and VraSR

**Fig. 9:**
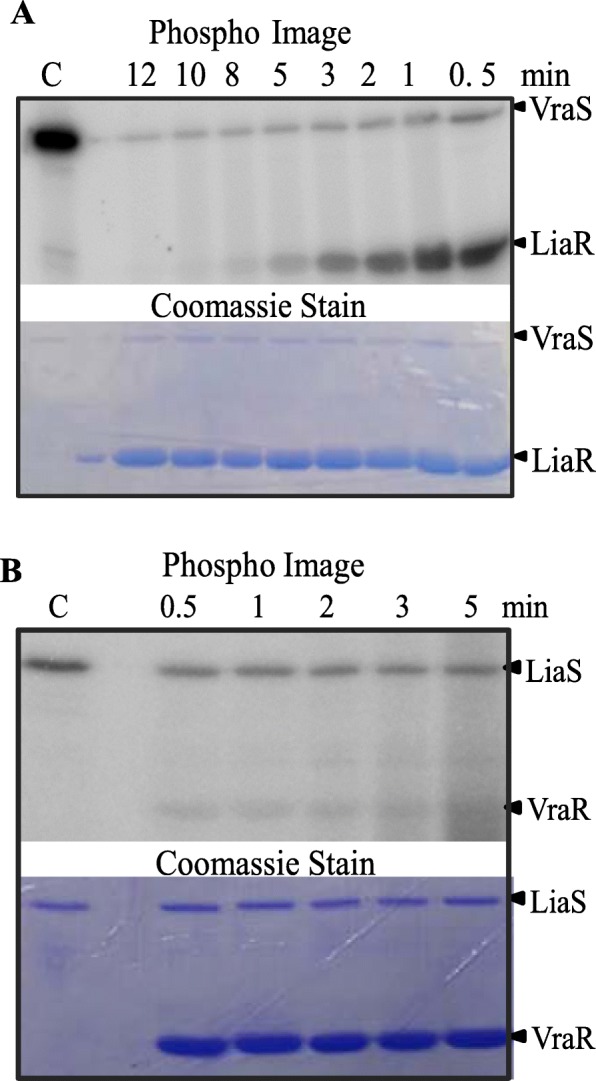
Cross-talk between non-cognate pairs VraS-LiaR (**a**) and LiaS-VraR (**b**). Phosphorylated VraS (or LiaS) at 5 μM was added to LiaR (or VraR) at 25 μM. The reaction mixtures were incubated at different time intervals at room temperature. The reactions were stopped by adding the SDS-PAGE loading dye. Samples were resolved by a 15% SDS-PAGE. The gels were exposed to a phosphor screen and imaged by a Typhoon Trio+ imager

The sequence alignments of LiaR and VraR, and LiaS and VraS showed that each pair of proteins share 52 and 34% sequence identity, respectively. Their involvement in signaling and regulation of the stress response to cell envelope damage led us to inquire whether they can cross-talk, i.e. whether LiaS can phosphorylate VraR, and whether VraS can phosphorylate LiaR. In addition, we investigated if LiaR could recognize the *vraSR* promoter and VraR could bind to the *liaI* promoter. These studies showed that VraS phosphorylates LiaR rapidly and efficiently, as it does with its cognate RR. By contrast, the efficiency of the phosphotransfer from LiaS to VraR was low, and it was similar to the way LiaS interacted with LiaR (Fig. 9a,b). The observation that each HK, VraS and LiaS, interacted with their non-cognate RR in the same way as each did with their cognate RR suggests that the structural conformation that each RR presents to these kinases is the same, i.e. LiaR and VraR are likely to share the same tertiary structure of their RDs.

**Fig. 10:**
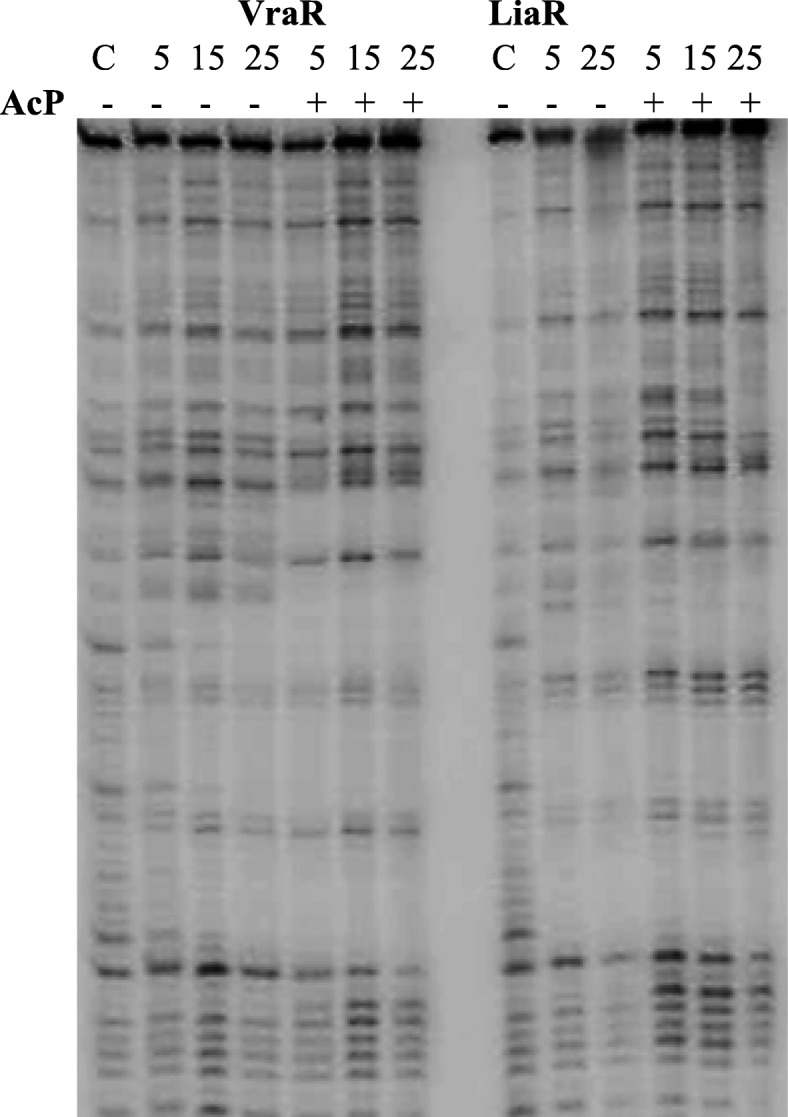
DNase I footprint analysis of the *vraSR* promoter with LiaR and VraR. The DNA fragment was labeled at the 5′-end of the top strand with [γ-^32^P]-ATP. The protein concentration is in micromolar

**Fig. 11:**
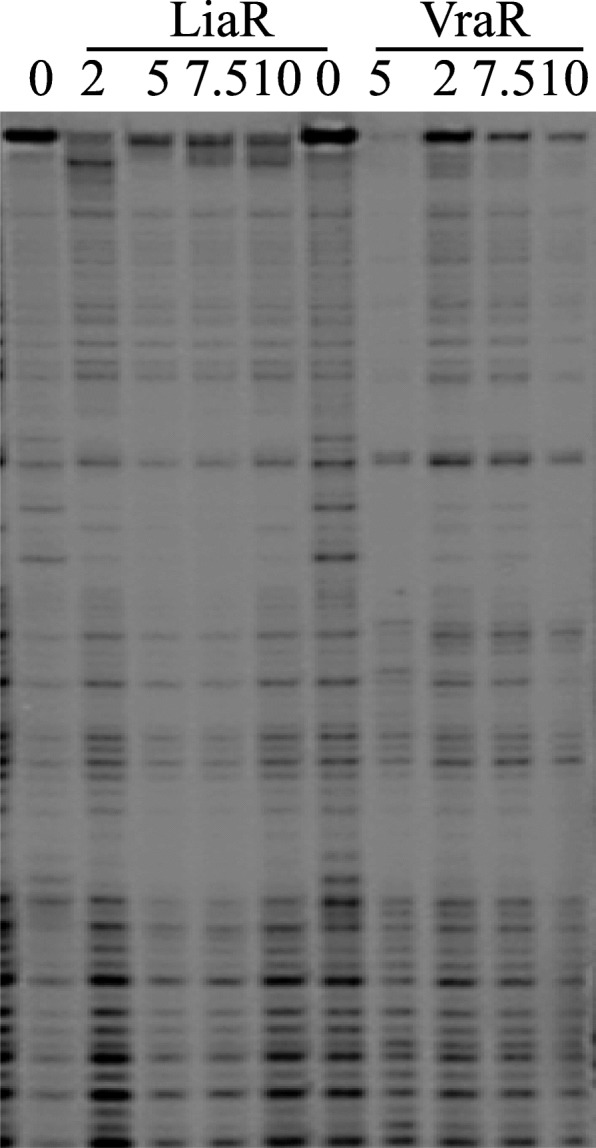
DNase I footprint analysis of P_*liaI*_ with LiaR and VraR. The DNA fragment was labeled the 5′-end of the top strand with [γ-^32^P]-ATP. The protein concentration is in micromolar

Furthermore, LiaR bound to the *vraSR* promoter similarly to VraR, that is, the unphosphorylated LiaR recognized the VraR primary-binding site and the phosphorylated LiaR recognized the VraR secondary-binding site on the *vraSR* promoter, albeit the binding affinities of LiaR for these two sites was weaker than VraR (Fig. 10). The DNase I footprinting experiments showed that VraR also bound to P_LiaI_ similarly to LiaR (Fig. 11). These observations suggest that the similarity in the amino acids sequence between these two ED extends to their DNA-binding modes. The difference in their binding affinities for their non-target promoters suggests that, in spite of conservation of their ED tertiary structures, small differences in their amino acid sequences are sufficient to refine the sequence specificity of these domains.

## Discussion

We undertook a close-up view of the LiaRS-mediated signal-transduction process in *B. subtilis* by characterizing the two molecular players, LiaS and LiaR, through a biochemical approach. The cell envelope stress response in *B. subtilis* is mediated by a three-component system, LiaFSR. LiaF, a repressor of the LiaS kinase activity was not considered in this study, since our primary focus was to characterize LiaS and LiaR as bona fide HK and RR, respectively, and compare them with other homolog two-component systems.

**Fig. 12:**
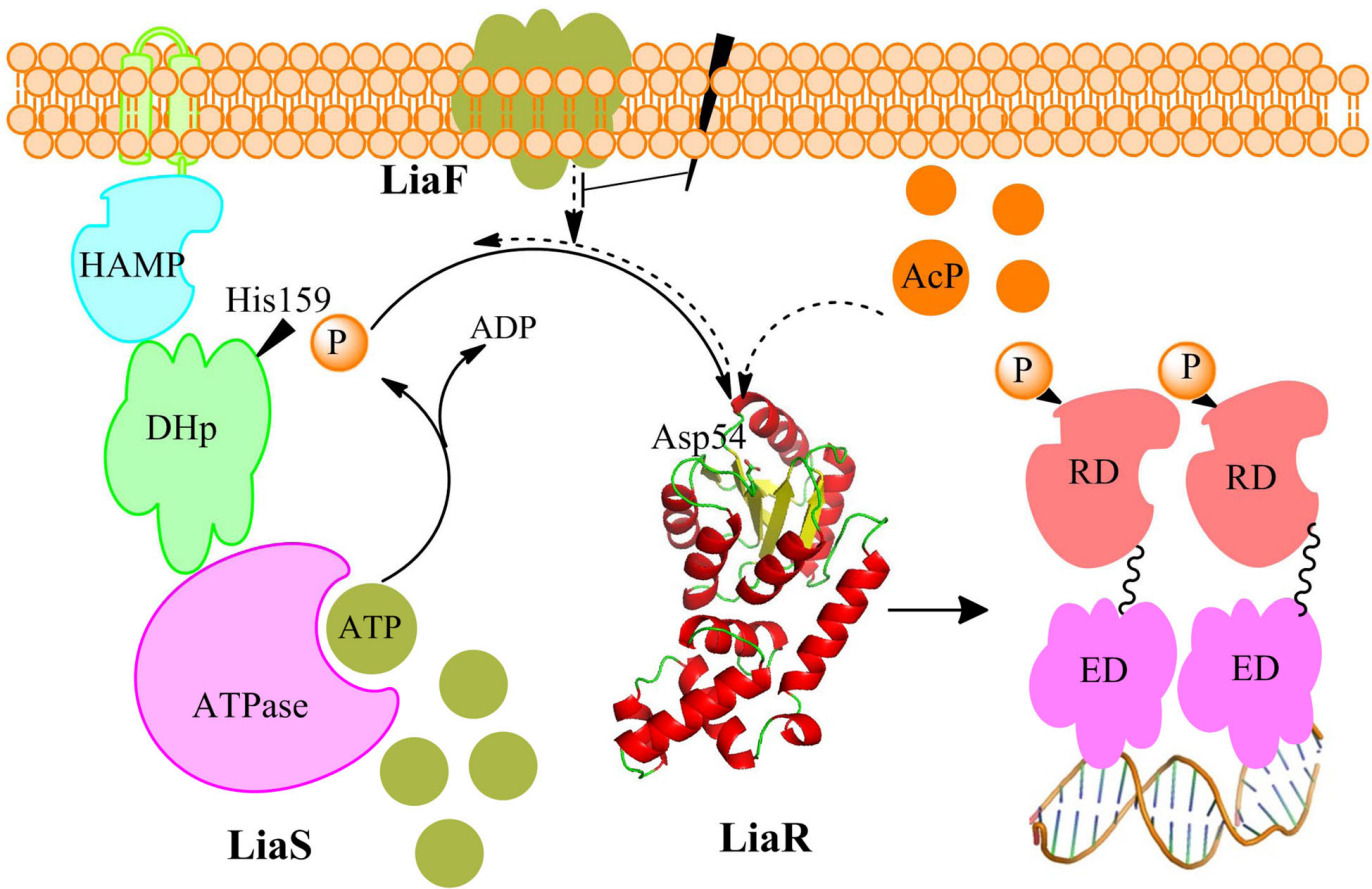
The LiaRS signal transduction pathway in *B. subtilis*, reflecting the findings in this study. The cell membrane disruption is represented by a solid black irregular shape. The solid arrows illustrate the processes that occur in the presence of the stimulus, and the dashed ones illustrate the processes that occur in the absence of stress; the inhibitory effect of LiaF on the phosphatase activity of LiaS is represented with a dashed arrow. The length of each arrow is intended to illustrate, in relative terms, the rate of the process that is represents

Our study shows that LiaS is a bifunctional enzyme with autokinase, and phosphatase activities toward LiaR (Fig. 12). In addition, we found that unphosphorylated LiaR exists as a mixture of monomers/dimers in our protein solutions and is capable of catalyzing its own phosphorylation in the presence of acetyl phosphate. The dimerization interface of LiaR is hosted at RD as in the case of many NarL/FixJ family of proteins [28, 38, 39, 41]. Furthermore, our results demonstrate that the dimerization of LiaR at RD is essential for the strong interaction of LiaR with P_*liaI*_. For the first time, we mapped the LiaR-binding sequence to P_*liaI*_ and show the significance of LiaR phosphorylation for its interaction with DNA. Lastly, the cross-talk between LiaRS and VraSR demonstrates the conservation of domain organization of HK and RR, and shows that in spite of the similarities in structure the slight differences in their amino acid sequences are sufficient to dictate differences in the behavior of these proteins in accordance with the needs of the cell.

### The kinetics of the autokinase activity of LiaS justifies the in vivo observation of molar excess of LiaS over LiaR

In this study we show that LiaS is capable of undergoing autophosphorylation, but at a slower rate than VraS. In addition, LiaS is capable of transferring the phosphoryl group to its cognate RR. However, the kinetics of this latter process was also slower in comparison to the VraS-mediated phosphotransfer process, measured under the same experimental conditions. Interestingly, VraS phosphorylated LiaR faster than LiaS. Taken together, we interpreted these observations as an indication that LiaS may be intrinsically less efficient than VraS in the signal transduction process. The lower efficiency of LiaS in the phosphotransfer process could also be due to a weak binding interaction between LiaS and LiaR. Indeed, the pull down experiments showed that LiaR could not be pulled down by LiaS (Additional file 9: Figure S9). The slower kinetics of LiaS autophosphorylation, the weak interaction between LiaS and LiaR, and the slow rate of the phosphotransfer process provides an explanation for the significance of the unusual 4:1 stoichiometry identified in vivo between LiaS and LiaR [14]. Although *B. subtilis* could have also encountered this disadvantage by increasing the LiaR concentration above that of LiaS, this strategy would not have been beneficial to the cell due to the kinetics of the LiaR phosphorylation and dephosphorylation processes and the tight interaction between LiaR and P_*liaI*_. For one, the signal-transduction processes would have been slow to be relevant in vivo [23], and in addition it could have led to a higher background expression level of the *liaIHFSR* locus, which in turn could have led to a constitutively “ON” state for this signaling pathway. Hence our kinetic data ultimately provide a reason for the unusual stoichiometry described for *B. subtilis*.

### Kinetics of the LiaS-dependent dephosphorylation of LiaR and LiaR phosphorylation by acetyl phosphate requires a molar excess of LiaS over LiaR in vivo

The phosphatase activity of LiaS toward LiaR-P is five-fold slower than that reported for VraS toward VraR-P [35]. This difference in the phosphatase activities between these two kinases is also reflected in the interaction with their non-cognate RR, i.e. VraS dephosphorylates LiaR faster than LiaS, suggesting that LiaS is intrinsically less efficient as a phosphatase. The poor phosphatase activity of LiaS may explain why LiaF is required to keep the LiaRS-mediated signal transduction pathway OFF in the absence of stimulus—it may enhance the phosphatase activity of LiaS. Indeed, deletion of *liaF* led to a constitutive activation of *lia* locus [15]. Accordingly, the poor phosphatase activity of LiaS in the absence of LiaF – as is the case in experimental conditions – explains the constitutive ON behavior of an overexpressed *liaS* mutant [14]. Efforts to clone *liaF* in *E. coli* for the purpose of isolating the protein have been unsuccessful thus hampering our efforts to characterize in vitro the mode of interaction between LiaF and LiaS [14].

Furthermore, while the rate constant of VraR dephosphorylation by VraS is 200-fold faster than the rate constant of VraR phosphorylation by acetyl phosphate [35], the rate constant of LiaR dephosphorylation by LiaS is only 40-fold faster than the rate of LiaR phosphorylation by acetyl phosphate. The difference in the HK-dependent dephosphorylation and the acetyl phosphate-dependent phosphorylation rate constants between these two phosphorylation-dependent signaling pathways (VraSR versus LiaRS) suggests that LiaR might undergo phosphorylation by acetyl phosphate and this species may be long lived under high concentrations of acetyl phosphate in the cell [42]. Schrecke et al. showed that the *liaS::kan* strain of *B. subtilis*, which overproduces LiaR as a result of a polar effect on the downstream genes upon *liaS* deletion by insertion of the kanamycin cassette, displayed a strong induction of the *lia* locus that was completely dependent on acetyl phosphate [14]. Hence, for *B. subtilis* to have tight control over the phosphorylation state of LiaR, the cells should produce LiaS at concentrations higher than LiaR, and, moreover, over-produce LiaF to ensure that LiaS operates as a phosphatase in the absence of stimulus.

### LiaR interacts very strongly with the liaI promoter

The unphosphorylated LiaR protein bound to P_*liaI*_ at a concentration as low as 200 nM. This is a rather high binding affinity that is not reported for other LiaR homologs. The high binding affinity of the unphosphorylated LiaR for P_*liaI*_ suggests that under uninduced conditions, when the expression of *lia* locus should be switched off, a few molecules of LiaR are sufficient to provide a low background expression of the operon. In addition, the tight binding of LiaR to P_*liaI*_ aligns well with the stoichiometry found for LiaS/LiaR in vivo.

The phosphorylation of LiaR with acetyl phosphate expanded the DNA binding region upstream of the primary LiaR-binding site, spanning the residues from − 89 to − 76. However, phosphorylation of LiaR did not have a large effect on the binding affinity of LiaR for its target promoter. The latter effect could be due to the low amount of the LiaR phosphorylated species in our protein preparations. Curiously, the LiaRD54A variant did not show an expansion of binding to P_*liaI*_ beyond − 76 position, even at high LiaRD54A concentration, an indication that expansion of the LiaR protection region on P_*liaI*_ is phosphorylation-dependent. Earlier work by Mascher et al. showed that the P_*liaI*_ region from − 74 to + 97 was sufficient to provide the highest level of *liaI* locus induction in the presence of bacitracin [8]. It is likely, that the expansion of the DNase I protection from − 89 to − 76 on P_*lia*_*,* upon LiaR phosphorylation, could be due to the formation of oligomers of LiaR beyond the dimers, such as tetramers which were seen in the crystal structures of other LiaR homologs such as VraR [41], *Efm* LiaR [38], and *Efc* LiaR [39]. Thus, as the phosphorylation of LiaR leads to more dimers, which in turn could lead to the formation of tetramers and their sequestration to the least conserved LiaR-binding sequence of P_*liaI*,_ (the secondary binding site), this could result in hindering of the DNase I activity at this site of the promoter. Hence, it is plausible that expansion of the DNA-binding region by LiaR-P beyond the − 74 position on P_*liaI*_ may not be relevant in vivo. Rather, the phosphorylation of LiaR may serve to increase the binding affinity of LiaR to its promoter and possibly to introduce bending of the DNA, as the appearance of the hypersensitive site at − 79 may suggest.

The recognition of LiaR-binding sequence on P_*liaI*_ by VraR and the VraR-binding sequence on the *vraSR* promoter by LiaR suggests that LiaR homologs may share a common ancestral protein that recognized a similar DNA-binding sequence. However, these proteins, together with the promoter regions of their respective loci, evolved to adapt different transcriptional regulatory mechanisms to better meet the needs of the cell [40].

## Conclusions

Our study shows that LiaS is a bi-functional enzyme with both autokinase and phosphatase activities. The slight deficiency of LiaS as a phosphatase and the considerable fast rate of LiaR phosphorylation by acetyl phosphate (Fig. 12) could have made this TCS vulnerable to nonspecific signals. However, these deficiencies are overcome by a higher concentration of LiaS over LiaR in the cell and a tight control of LiaF over the LiaS phosphatase activity, also enabled by a surplus of LiaF over LiaS, in the absence of stimulus [14]. Moreover, the strong interaction of LiaR with P_*liaI*_ provides the cell with sufficient copies of LiaRS to guard the cell envelope in the absence of stress. Lastly, in spite of the similarities among the LiaRS homologs in particular, and among TCS in bacteria in general, our study demonstrates that TCS evolve to adapt to the needs of a particular species. The adaptation of TCS may involve tweaking of the dynamics of the interactions between HK and RR and/or of the kinetics of the phosphorylation-mediated processes.

## Methods

Chemicals and antibiotics were purchased from Sigma (Oakville, Canada) or ThermoFisher (Whitby, Canada), unless otherwise stated. Chromatography media and columns were purchased from GE Healthcare (Quebec, Canada). Growth media were purchased from ThermoFisher. *Escherichia coli* strains, NovaBlue and BL21(DE3), and cloning and expression plasmids were purchased from EMD Biosciences (New Jersey, USA). The pGEX-4 T vector was purchased from GE Healthcare (Quebec, Canada). Restriction enzymes were obtained from New England Biolabs Canada (Pickering, Canada) or ThermoFisher. The [γ-^32^P]-ATP was purchased from Perkin Elmer LAS Canada Inc. (Toronto, Canada) or GE Healthcare. The Proteo Extract All-in-One Trypsin Digestion Kit was purchased from EMD Bioscience. Chromosomal DNA of the *Bacillus subtilis* strain 168 was acquired from Cedarlane. Oligonucleotides were acquired from Sigma (Canada).

### Cloning the cytoplasmic domain of the LiaS protein, namely *liaS*^126^, fused to the C-terminus of the GST protein

The following direct and reverse primers were used to amplify the nucleic acid sequence that encodes the cytoplasmic region (amino acids 126–360) of *B. subtilis liaS*: Dir: 5′- CAT*GGATCC*CGATTGGCCAGAGATCTTC-3′ and Rev.: 5′-ACG*CCCG GGTTA*TCAATCAA TAATACTCGAATC-3′. The primers contain the restriction enzymes *BamH*I and *Sma*I (the italicized sequence), respectively. We used chromosomal DNA of *B. subtilis* 168 as the template for *liaS* amplification. The amplified gene is referred to as *liaS*^126^. The amplified gene (708 bp) was digested with the above restriction enzymes and subsequently cloned into the corresponding restriction sites of the pGEX-4 T-1 vector (GE Healthcare) to fuse *liaS*^126^ to the C-terminus of the GST protein. The resulting construct pGEX-4 T-1::*liaS*^126^ was introduced into *E. coli* BL21(DE3). The correctness of the *liaS*^126^ insertion into pGEX-4 T-1 was confirmed by DNA sequencing (The Centre for Applied Genomics, The Hospital for Sick Kids, Toronto, Canada). Hereon the LiaS chimeric construct is referred to as LiaS.

### Cloning of the *liaR* gene encoding the full-length LiaR protein

Chromosomal DNA of *B. subtilis* 168 was used as a template for the amplification of the *liaR* gene encoding for the full length and tagless LiaR protein, corresponding to the amino acids 1 to 211. The following primers were used for the amplification of *liaR*: Dir: 5′-ACG*CATATG*ATGATTCGAGTATT ATTGAT-3′ and Rev.: 5′-ACG*AAGCTT*CTAATTCACGAGATGATTT-3′. The primers contained the restriction sites for *Nde*I and *Hind*III (italicized sequences), respectively. The amplicon was digested with *Nde*I and *Hind*III and ligated into pET26b vector between the *Nde*I and *Hind*III restriction sites. The resulting construct, pET26b::*liaR,* was introduced into *E. coli* BL21(DE3). The correctness of the *liaR* cloning to pET26b was confirmed by DNA sequencing (The Center for Applied Genomics, The Hospital for Sick Kids, Toronto, Canada).

### Cloning of *liaR*^*N*^ and *liaR*^*C*^, encoding the N-terminal and C-terminal domains of LiaR

The Gly-131 residue of LiaR (GGA) was mutated to a stop codon (TGA) in order to clone the N-terminal domain of LiaR (*liaR*^N^). The mutation was carried out using Site-Directed mutagenesis kit (ThermoFisher) with the following primers Dir: 5′-AAAGTGGCG*TGA*AAAGTATTATCC AGGCT-3′ and Rev.: 5′-AGCCTGGATAAT*ACT*TTTCACGCCACTTT − 3′ (the mutated codon is italicized). The pET26b::*liaR* vector was used as the template. The successful introduction of the stop codon was verified by DNA sequencing. The resulting pET26b::*liaR*^*N*^ construct was used for transformation of *E. coli* BL21 (DE3).

The cloning strategy for the C-terminal domain of LiaR (*liaR*^C^) was similar to that employed for cloning of the full-length LiaR. The nucleotide sequence that encodes ED, spanning the residues 140–211 (the sequence was determined by ExPASy tools), was amplified using pET26b::*liaR as the template* and the following primers: Dir: 5′- ACG*CATATG*TCAGGTGA AAACGC-3′ and Rev.: 5′-ACG*AAGCTT*CTAATTCACGAGATGATTT-3′ (the restriction sites *Nde*I and *Hind*III are italicized). The subsequent construct, pET26b:*:liaR*^*C*^*,* was introduced into *E. coli* BL21(DE3). The correctness of the *liaR*^*C*^ cloning was verified by DNA sequencing.

### Mutagenesis on *liaS* and *liaR*

We carried out the mutation of the conserved phosphorylation site on LiaS, His154 (CAT) to Ala (GCT) substitution, and on LiaR, Asp54 (GAT) to Ala (GCC) substitution. The mutagenesis on *liaS* and *liaR* was carried out using the Single-Site Directed mutagenesis kit (ThermoFisher). In the case of mutagenesis on *liaS*, the pGEX-4 T1::*liaS*^*126*^ vector was used as a template and the following primers were used as the mutagenesis primers: Dir: 5′-CCAGAGATCTT*GCT*GATGCGGTCAG-3′ and Rev.: 5′-CTGACCGCATC*AGC*AAG ATCTCTG G-3′ (the mutation site is italicized). In the case of mutagenesis on *liaR*, the pET26b::*liaR* vector was used as a template and the following primers were used as the mutagenesis primers: Dir: 5′-GATGTCATTTTAATG*GCC*CTTGTCATGG-3′ and Rev.: 5′-CC ATGACAAG*GGC*CATTAAAATGACATC-3′ (the mutation site is italicized). The successful mutations were confirmed by DNA sequencing.

### Purification of the target proteins

The expression of *liaS* and its variants, and production and isolation of their respective proteins were carried out as described previously by Belcheva and Golemi-Kotra [35]. The expression of *liaR*, its variants and its domains were carried out as previously described by Belcheva and Golemi-Kotra [35]. The purification of LiaR or LiaR^C^ was performed as previously described for VraR with a few modifications [35]. Briefly, the cell pellet was resuspended in the 50 mM Tris-HCl, pH 7.0 buffer, supplemented with 5 mM MgCl_2_, and sonicated to liberate the soluble proteins. Cell debris was removed by centrifugation at 21,000×g, for 1 h at 4 °C. The resulting supernatant was loaded onto a DEAE-Sepharose column and LiaR was eluted using a linear gradient of 500 mM Tris-HCl, pH 7.0 buffer, supplemented with 5 mM MgCl_2_. The protein containing fractions were examined by 15% SDS PAGE. LiaR containing fractions were concentrated using an Amicon stirred cell (MWCO, 5 kD). The protein was loaded onto the Heparin-Sepharose column that was equilibrated with 50 mM Tris-HCl, pH 7.0 buffer, supplemented with 5 mM MgCl_2_. The protein was eluted using a linear gradient of 500 mM Tris-HCl, pH 7.0 buffer, supplemented with 5 mM MgCl_2._ The protein containing fractions were analyzed using a 15% SDS-PAGE. The fractions containing LiaR were combined and concentrated using Amicon centrifugal filter tubes (MWCO 3 kD). In the case of purification of LiaR^N^, as a second purification step, we used a Sephacryl S-200 HiPrep 26–60 size exclusion column that was equilibrated with 50 mM Tris, pH 7.0 buffer, supplemented with 100 mM KCl and 5 mM MgCl_2_. Protein containing fractions were analyzed by 20% SDS-PAGE. The fractions containing LiaR^N^ were combined and concentrated using Amicon centrifugal filter tubes (MWCO 3 kD).

The identity of the purified proteins was confirmed by either in-gel trypsin digestion followed by MALDI (Center for Mass Spectrometry Research, York University) and/or electrospray ionization mass spectrometry. The proper folding of our protein preparations were assessed by comparing the CD spectra and the thermal melting curves with those obtained from our previous studies on the VraSR, GraSR and LytSR systems [30, 35, 43].

### Circular dichroism (CD) spectroscopy and thermal melting of the target proteins

Typically, the CD spectrum of the target protein was obtained by preparing a 10 μM protein solution in 50 mM Tris-HCL, pH 7.0 buffer, supplemented with 5 mM MgCl_2_. The CD spectrum was recorded from 200 to 260 nm using a JascoJ-810 instrument (path length of the cuvette was 0.1 cm). Thermal melting curves were obtained by measuring the CD signal at 222 nm from 25 °C to 90 °C, at a rate of 5 °C/min.

### In vitro autophosphorylation of LiaS

The in vitro autophosphorylation of LiaS was carried out at ATP concentrations ranging from 10 μM to 250 μM (each concentration was repeated two times). Different ATP concentrations were prepared by mixing radiolabeled [γ-^32^P]-ATP and non-radiolabeled ATP (a 1:9 ratio was maintained). Typically, LiaS (5 μM) in the phosphorylation buffer (PB: 50 mM Tris, pH 7.4 buffer, 50 mM KCl, 5 mM MgCl_2_) supplemented with 10 mM CaCl_2_ was incubated with [γ-^32^P]-ATP (250 Ci) at different time intervals (at 25 °C). The reactions were quenched by addition of SDS sample buffer dye. Samples were analyzed by 12.5% SDS-PAGE. The gel was incubated on a phosphor screen for 2 h. The screen was scanned using a Typhoon Trio^+^ instrument. The quantification of phosphorylation was done by ImageJ (NIH). The time dependent data were fitted by a first-order rate equation using Erithacus GraFit software (version 5.0.10).

### Phosphotransfer reaction

Phosphorylation of LiaS was carried out as described above. The excess of [γ-^32^P]-ATP (3000 Ci/mmol) was removed using a desalting column (Zeba™, Pierce). Then the phosphorylated LiaS (4 μM) was added to LiaR (20 μM) in the PB buffer (50 mM Tris, pH 7.4, 50 mM KCl, 20 mM MgCl_2_). The reaction mixture was incubated at 25 °C and samples of 10 μl were removed at different time intervals and quenched with SDS sample buffer. The quenched reactions were analyzed by 15% SDS-PAGE. The extent of the phosphotransfer was monitored by exposing gels to a phosphor screen (GE HealthCare) for 2 h and imaging them using a Typhoon Trio+ variable-mode imager (GE HealthCare). The gels were stained with Coomassie Blue to assess the protein amounts across the different time-point reaction mixtures. The phosphor images of the gels were analyzed by ImageJ (NIH). These experiments were repeated two times.

### Phosphorylation of LiaR by acetyl phosphate

We used acetyl phosphate to investigate the phosphorylation of LiaR by small molecule phosphate donors. Briefly, LiaR (30 μM) in the PB buffer was incubated with lithium potassium acetyl phosphate (50 mM). The reaction mixture was incubated at 37 °C at different time intervals. The extent of phosphorylation was investigated by 15% SDS-PAGE, containing acrylamide-pedant Phos-tag™AAL-107 at 50 μM (Cedarlane) [44]. These experiments were repeated two times.

### Investigation of the oligomerization state of LiaR, and its stand-alone domains

The oligomerization state of LiaR and LiaR^N^ prior to and following its phosphorylation was examined by native-PAGE (continuous gels). Solutions of 10, 20 and 30 μM of LiaR (or LiaR^N^, LiaR^C^) in the PB buffer, supplemented with 20 mM MgCl_2_, were supplemented with 50 mM acetyl phosphate and incubated for 1 h at 37 °C. Typically, 20 μl aliquots were removed and quenched by native sample buffer. The gels were stained with Coomassie Blue to visualize the protein bands. LiaR^C^ was analyzed on an acidic native-PAGE.

### Electromobility shift assays

The DNA-binding affinity of LiaR to the *liaI* promoter (P_*liaI*_) was analyzed by electromobility shift assays (EMSA). Typically, P_*liaI*_ at 2 ng/μl was 5′-end labeled with [γ- ^32^P]-ATP (3000 Ci/mmol) using T4 polynucleotide kinase. The binding reactions (20 μl) were prepared in the binding buffer (10 mM Tris, pH 7.5, 50 mM KCl, and 1 mM DTT) supplemented with 5 mM MgCl_2_, 10 ng of herring sperm DNA, and 2.5% glycerol. The DNA was mixed with unphosphorylated and phosphorylated LiaR at concentrations varying from 0 to 10 μM. The reaction mixtures were incubated at 25 °C for 30 min and resolved by 9% native-PAGE. The dried gels were exposed to phosphor screens (GE HealthCare) and scanned by a Typhoon Trio^+^ variable-mode imager (GE HealthCare). The gel images were analyzed by ImageJ (NIH). The dissociation constant, *K*_d_, was determined as the concentration of the protein required to shift 50% of the DNA. These experiments were repeated at least three times.

### DNase I footprinting experiments

P_*liaI*_ spanning the nucleotide sequence from − 162 to + 31 was amplified using the primers Dir:5′-GAAAGGGAAGCAAGTGTTCATCTGTAAAG-3′ and Rev.:5′- TTCATGCAGATCCTCCTTTCGTTTT-3′. The DNase I footprinting was carried out as previously described [35].

## Supplementary information

**Additional file 1.** Purification of LiaS and LiaR.

**Additional file 2.** Purificaiton of LiaR^C^ and LiaR^N^.

**Additional file 3.** Autokinase activity of LiaS. Progress curve of time-dependent phosphorylation of LiaS, and SDS-PAGE analysis of LiaS and LiaSH159A phosphorylation.

**Additional file 4.** Phosphorylation of LiaR^N^ by acetyl phosphate.

**Additional file 5.** CD studies on LiaR and LiaRD54A variant.

**Additional file 6.** Phosphorylation of LiaRD54A by acetyl phosphate.

**Additional file 7.** Oligomerization studies on LiaR^N^.

**Additional file 8.** The DNA sequencing standard reactions.

**Additional file 9.** The pull-down experiments with GST-LiaS and LiaR.

## Data Availability

The datasets supporting the conclusions of this article are included within the article and/or additional supporting files. The raw data on time-dependence of LiaS and LiaR phosphorylation are available from the corresponding author upon reasonable request.

## Abbreviations

LiaRS: Lipid II cycle interfering antibiotic response regulator and sensor
SDS: Sodium dodecyl sulphate
PAGE: Polyacrylamide gel electrophoresis
EMSA: Electromobility shift assay
ATP: Adenosine triphosphate
CD: Circular dichroism
PCR: Polymerase chain reaction
