# Supplementary material for: Low phosphatase activity of LiaS and strong LiaR-DNA affinity explain the unusual LiaS to LiaR in vivo stoichiometry
Source: BMC Microbiol. 2020 Apr 29;20:104. doi: 10.1186/s12866-020-01796-6 (PMC7191749; doi:10.1186/s12866-020-01796-6)
Supplement: Supplementary file 1 — Additional file 1. Purification of LiaS and LiaR. [file 12866_2020_1796_MOESM1_ESM.pdf]

Addional File 1

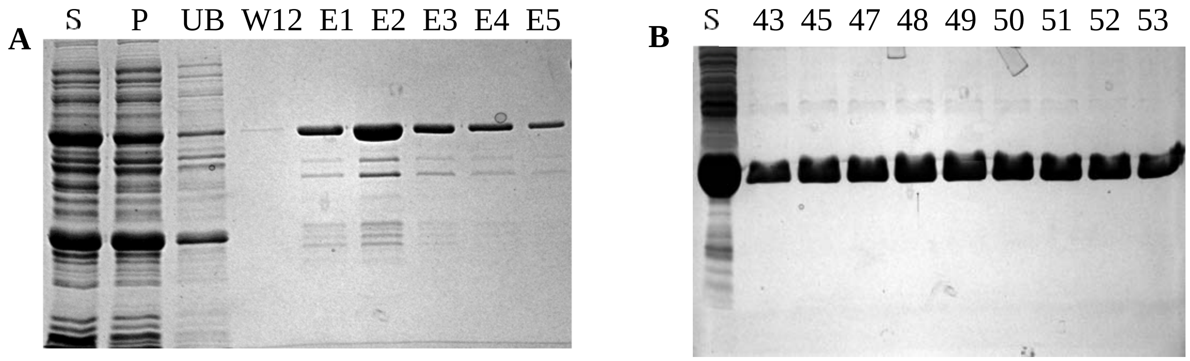

Fig. S1. (A) Purification of GST-LiaS (LiaS spanning the amino acids 126 to 360). This is a coomassie stained 12.5% SDS-PAGE. Lane 1, is cell extract obtained after the sonication of *E. coli* BL21(DE3) induced with IPTG. Lane 2, is cell debris collected after the sonication of the *E. coli* BL21(DE3) induced with IPTG. Lane 3, is the protein fraction collected during the washing of the GST-agarose column. Lanes 4-10, are protein fractions collected during the elution of the GST-agarose column with buffer supplemented with 10 mM glutathione. (B) LiaR fractions collected at the last step of purification (Heparin column). Lane 1 (denoted as S) is the protein extract collected at the first step of purification (DEAE column), which was subsequently loaded on the heparin column.
