# Supplementary material for: Low phosphatase activity of LiaS and strong LiaR-DNA affinity explain the unusual LiaS to LiaR in vivo stoichiometry
Source: BMC Microbiol. 2020 Apr 29;20:104. doi: 10.1186/s12866-020-01796-6 (PMC7191749; doi:10.1186/s12866-020-01796-6)
Supplement: Supplementary file 2 — Additional file 2. Purificaiton of LiaRC and LiaRN. [file 12866_2020_1796_MOESM2_ESM.pdf]

Additional File 2

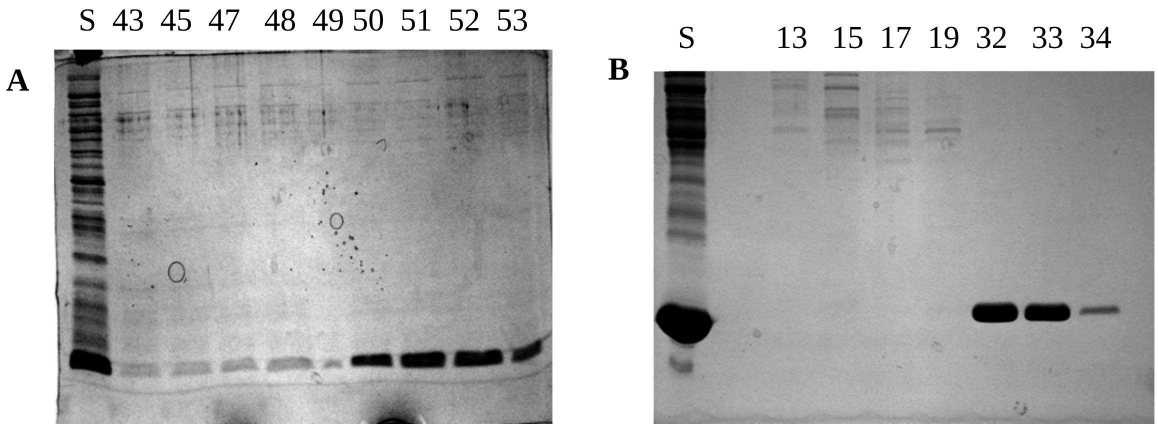

Fig. S2. (A) LiaR^{C} fractions collected at the last step of purification (Heparin column; Lanes 2 to 10). Lane 1 (denoted as S) is the protein extract collected at the first step of purification (DEAE column), which was subsequently loaded on to the heparin column. (B) The LiaR^{N} fractions collected at the last step of purification, the Sephacryl S-200 HiPrep column. A 20% SDS-PAGE shows in Lane 1 the cell extract after sonication of *E. coli* BL21(DE3) cells induced with IPTG, in Lanes 3-10 the fractions 13-34 collected during protein elution from the Sephacryl S-200 HiPrep column.
