# Supplementary material for: Low phosphatase activity of LiaS and strong LiaR-DNA affinity explain the unusual LiaS to LiaR in vivo stoichiometry
Source: BMC Microbiol. 2020 Apr 29;20:104. doi: 10.1186/s12866-020-01796-6 (PMC7191749; doi:10.1186/s12866-020-01796-6)
Supplement: Supplementary file 3 — Additional file 3. Autokinase activity of LiaS. Progress curve of time-dependent phosphorylation of LiaS, and SDS-PAGE analysis of LiaS and LiaSH159A phosphorylation. [file 12866_2020_1796_MOESM3_ESM.pdf]

Additional File 3

A

B

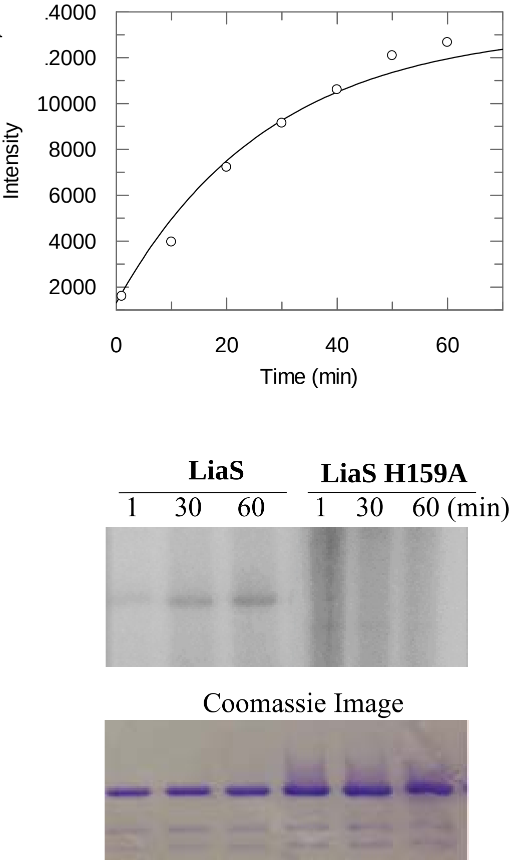

Fig. S3. Autokinase activity of LiaS. (A) LiaS at 5 μM was incubated with 250 uM [γ-^{32}P] ATP in PB at 25°C. The reaction was quenched at different time intervals and samples were analyzed by a 12.5% SDS-PAGE. Phosphorylation of LiaS with γ-^{32}P was quantified by exposing the SDS-PAGE to a phosphor screen and measure the densitometry of each protein band (the Intesity values (Y-axis) in the graph) using ImageJ. (B) Autokinase kinase activities of LiaS and LiaSH159. LiaS at 5 μM was incubated with 25 uM [γ-^{32}P] ATP in PB at 25°C. The reaction was quenched at different time intervals and samples were analyzed by a 12.5% SDS-PAGE. The SDS-PAGE was scanned with a phosphor screen to quantify the incorporation of γ-^{32}P to LiaS (top panel). The bottom panel is a coomassie stain of the SDS-PAGE to ensure equal loading of the samples.
