# Supplementary material for: Low phosphatase activity of LiaS and strong LiaR-DNA affinity explain the unusual LiaS to LiaR in vivo stoichiometry
Source: BMC Microbiol. 2020 Apr 29;20:104. doi: 10.1186/s12866-020-01796-6 (PMC7191749; doi:10.1186/s12866-020-01796-6)
Supplement: Supplementary file 4 — Additional file 4. Phosphorylation of LiaRN by acetyl phosphate. [file 12866_2020_1796_MOESM4_ESM.pdf]

Additional File 4

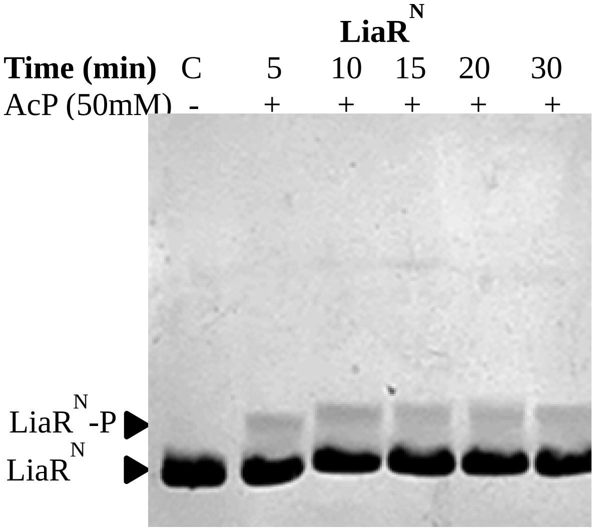

Fig. S4. Phosphorylation of LiaR^{N} by acetyl phosphate. LiaR^{N} at 30 µM was incubated with 50 mM acetyl phosphate in PB at different time intervals. The reaction was quenched by the addition of SDS-PAGE loading dye. Reaction samples were analyzed in a 18% SDS-PAGE containing Phospho-TagT^{M}. Gels were quantified by densitometry of bands using ImageJ.
