# Supplementary material for: Low phosphatase activity of LiaS and strong LiaR-DNA affinity explain the unusual LiaS to LiaR in vivo stoichiometry
Source: BMC Microbiol. 2020 Apr 29;20:104. doi: 10.1186/s12866-020-01796-6 (PMC7191749; doi:10.1186/s12866-020-01796-6)
Supplement: Supplementary file 5 — Additional file 5. CD studies on LiaR and LiaRD54A variant. [file 12866_2020_1796_MOESM5_ESM.pdf]

Additional File 5

A

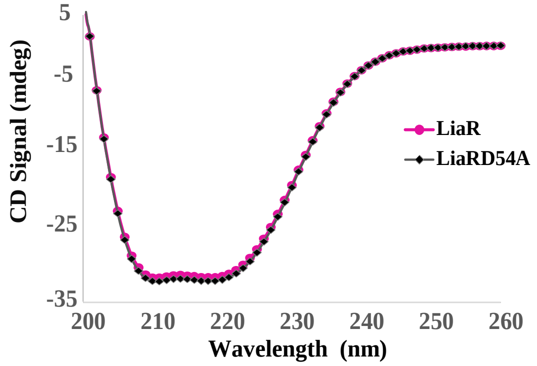

B

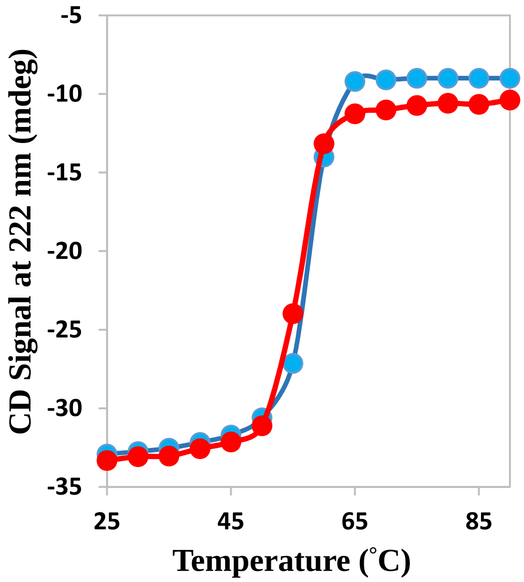

Fig. S5. CD studies on LiaR and LiaRD54A variant. (**A**) The CD spectra of LiaR and LiaRD54A. Briefly, each protein at 10 µM was prepared in 50 mM Tris, 5 mM $MgCl_2$ at pH 7.0. (**B**) The thermal melting of LiaR (blue dots) and LiaRD54A (red dots). The samples were prepared similarly as for panel (A).
