# Supplementary material for: Low phosphatase activity of LiaS and strong LiaR-DNA affinity explain the unusual LiaS to LiaR in vivo stoichiometry
Source: BMC Microbiol. 2020 Apr 29;20:104. doi: 10.1186/s12866-020-01796-6 (PMC7191749; doi:10.1186/s12866-020-01796-6)
Supplement: Supplementary file 6 — Additional file 6. Phosphorylation of LiaRD54A by acetyl phosphate. [file 12866_2020_1796_MOESM6_ESM.pdf]

Additional File 6

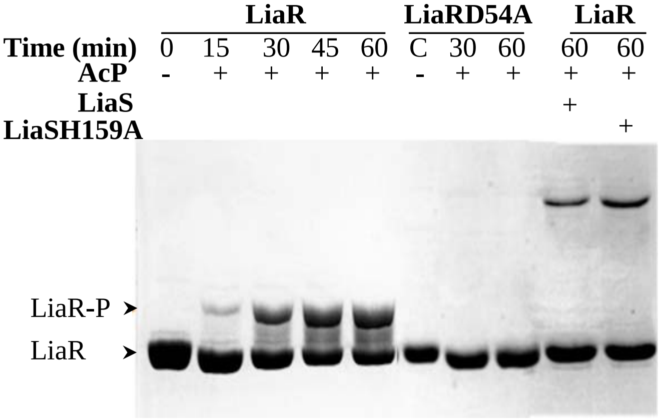

Fig. S6. Phosphorylation of LiaR and LiaRD54A by acetyl phosphate. LiaR or LiaRD54A at 30 µM was incubated with 50 mM acetyl phosphate in PB at different time intervals. The reaction was quenched by the addition of SDS-PAGE loading dye. The phosphatase activity of LiaS and LiaSH159A on phosphorylated LiaR was assessed by adding the respective LiaS protein to the reaction mixture of LiaR. Reaction samples were analyzed in a 12.5% SDS-PAGE containing Phospho-TagT^M. Gels were quantified using by densitometry of bands using ImageJ.
