# Supplementary material for: Low phosphatase activity of LiaS and strong LiaR-DNA affinity explain the unusual LiaS to LiaR in vivo stoichiometry
Source: BMC Microbiol. 2020 Apr 29;20:104. doi: 10.1186/s12866-020-01796-6 (PMC7191749; doi:10.1186/s12866-020-01796-6)
Supplement: Supplementary file 7 — Additional file 7. Oligomerization studies on LiaRN. [file 12866_2020_1796_MOESM7_ESM.pdf]

Additional File 7

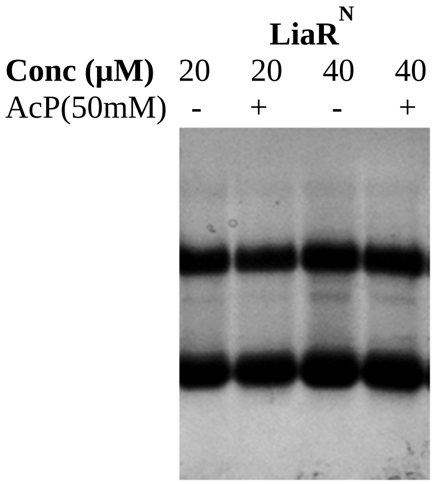

Fig. S7. Oligomerization studies on LiaR^N. The oligomerization state of LiaR^N was analyzed by a 15% native-PAGE. Samples with or without acetyl phosphate (AcP) were incubated for 1 h in PB, at room temperature, and were resolved by gel electrophoresis.
