# Supplementary material for: Low phosphatase activity of LiaS and strong LiaR-DNA affinity explain the unusual LiaS to LiaR in vivo stoichiometry
Source: BMC Microbiol. 2020 Apr 29;20:104. doi: 10.1186/s12866-020-01796-6 (PMC7191749; doi:10.1186/s12866-020-01796-6)
Supplement: Supplementary file 8 — Additional file 8. The DNA sequencing standard reactions. [file 12866_2020_1796_MOESM8_ESM.pdf]

Additional File 8

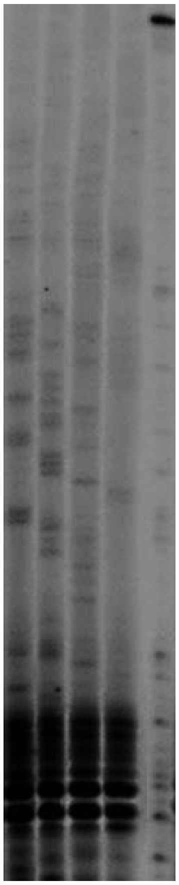

Fig. S8. Standard reactions using di-deoxynucleotides and P_{liaI} to determine the LiaR binding sequence in P_{liaI}. Lanes 1 to 4, are the standard reactions with the dideoxynucleotides ddCTP, ddGTP, ddTTP and ddATP, respectively, 5'[^{32}P]-end labelled forward primer, and P_{liaI} as a template. Lane 5, is the P_{liaI} digested with DNAse I. The DNA fragment was radiolabelled at the top strand (at the 5'-end).
