# Supplementary material for: Low phosphatase activity of LiaS and strong LiaR-DNA affinity explain the unusual LiaS to LiaR in vivo stoichiometry
Source: BMC Microbiol. 2020 Apr 29;20:104. doi: 10.1186/s12866-020-01796-6 (PMC7191749; doi:10.1186/s12866-020-01796-6)
Supplement: Supplementary file 9 — Additional file 9. The pull-down experiments with GST-LiaS and LiaR. [file 12866_2020_1796_MOESM9_ESM.pdf]

Additional File 9

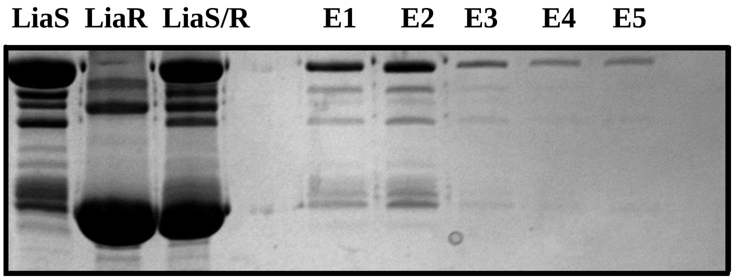

Fig. S9. Pull down assay for LiaS and LiaR where GST tagged LiaS was immobilized glutathione resin at room temperature. A 12.5% SDS gel showing LiaS, LiaR, and LiaS+LiaR used as MW controls. E1 to E5 indicate elution fractions collected with 10 mM reduced glutathione in 50 mM Tris (pH 8.0), after 5 minutes incubation. The proteins bands with MW close to LiaR were analyzed by in-gel trypsin digestion followed by mass-spectrometry to probe presen of LiaR. No LiaR was identified, hence we concluded LiaR was not pulled down in this experiments.
